# Inference with non-probability samples and survey data integration: a science mapping study

**DOI:** 10.1007/s40300-023-00243-6

**Published:** 2023-04-08

**Authors:** Camilla Salvatore

**Affiliations:** 1 Department of Economics, Management and Statistics (DEMS), University of Milano-Bicocca, Milan, Italy; 2 Faculty of Social and Behavioural Sciences, Universiteit Van Amsterdam, Amsterdam, The Netherlands

**Keywords:** Bibliometric analysis, Thematic analysis, Survey data integration, Nonprobability samples, New data sources

## Abstract

In recent years, survey data integration and inference based on non-probability samples have gained considerable attention. Because large probability-based samples can be cost-prohibitive in many instances, combining a probabilistic survey with auxiliary data is appealing to enhance inferences while reducing the survey costs. Also, as new data sources emerge, such as big data, inference and statistical data integration will face new challenges. This study aims to describe and understand the evolution of this research field over the years with an original approach based on text mining and bibliometric analysis. In order to retrieve the publications of interest (books, journal articles, proceedings, etc.), the Scopus database is considered. A collection of 1023 documents is analyzed. Through the use of such methodologies, it is possible to characterize the literature and identify contemporary research trends as well as potential directions for future investigation. We propose a research agenda along with a discussion of the research gaps which need to be addressed.

## Introduction

The field of survey research has experienced a profound transformation since the end of the 1990s due to the opportunity to use new data sources to make population inferences or to be integrated with traditional surveys [29]. Data integration is not new to survey researchers, who have already combined surveys based on probability-based samples (PS) with auxiliary data from censuses or administrative registers to enhance inference. However, as a result of technological progress and people’s changing interaction with technologies, a variety of new data sources have become available, and their use for inferential purposes poses new challenges as well as opportunities.

Probabilistic surveys are designed to provide unbiased, accurate, and reliable population statistics. However, in practice, unbiasedness can be undermined by various factors, such as non-coverage, nonresponse, and other sources of error, as described by the Total Survey Error (TSE) framework [13]. Since the early 1980s, nonresponse, in particular, has increased significantly, primarily because of an increase in non-contacts and refusals [60]. Consequently, a rethinking of incentives strategy and increased fieldwork efforts have raised survey costs to the point that many organizations can no longer undertake large and prohibitively expensive PS surveys.

Starting from the 2000s, volunteer web surveys and (big) digital trace data (textual data from social media, Google searches and maps, sensor data, etc.) have become popular data sources that can potentially replace or be integrated with traditional PS surveys. In general, they provide a more convenient and timely source of information for understanding complex social phenomena [48]. However, their non-probabilistic nature poses inferential and statistical challenges. The following paragraphs present three of these challenges, which will be discussed in more detail in Sect. 2.

The first challenge is selection bias that arises from the lack of a known selection mechanism and from the self-selection of individuals. Consequently, additional effort is required so that the estimates can be generalized. A second concern is the possibility that measurements of a particular construct may differ depending on the survey mode and characteristics of the auxiliary data sources. For instance, differences in measurement may arise when considering two surveys conducted in different modes (e.g., face-to-face vs. online) or one survey and a big data source (e.g., answers to a Likert scale vs. social media sentiment). As a third consideration, the quality of the data may also differ. Accordingly, ad-hoc quality and error frameworks need to be developed for each auxiliary source.

As a result of the above concerns, it is unlikely that data from non-probability samples (NPS) will replace traditional probabilistic surveys. However, supplementing a probabilistic survey with such auxiliary data is an appealing way to enhance inference while reducing the survey costs and respondent burden. The variety of these digital data requires more research on methodological aspects to address the statistical challenges mentioned above, as well as, applications to understand the potential benefits of building multi-source statistics. In particular, there are two main research streams [70]. The first stream of research focuses on inference based on NPS (addressing quality issues and correcting selection bias using PS surveys). The second research stream aims to statistically integrate NPS with PS surveys. In both cases, a central assumption is a high-quality PS survey.

This study aims to provide an overview of the current state of research in survey data integration and inference for non-probability samples. For this purpose, we analyze a selection of publications related to that topic using text mining and bibliometric techniques. In terms of a bibliographic database, we consider Scopus. This database allows the collection of document metadata such as the title, year of publication, journal, authors, and abstract. As opposed to other literature reviews, the originality of this study lies in the use of bibliometric and text mining tools. These tools allow us to analyze a greater number of papers, identifying current research trends, and to suggest future research directions.

The paper is organized as follows. Section 2 provides the literature background and the context of this study. The objectives of the research and the data are presented in Sect. 3. Section 4 describes the methodology. A detailed discussion of the results can be found in Sect. 5. In conclusion, Sect. 6 outlines a research agenda and identifies remaining research gaps to be addressed.

## Conceptual background

This section focuses on two aspects. Firstly, it describes the context of this work which is essential in order to critically evaluate the results of our study which will provide further insights. Secondly, it reviews the methodological literature in light of the three statistical challenges described in Sect. 1.

It is becoming increasingly common for researchers and statistical institutes to integrate data and make inferences based on non-probabilistic samples. As a complement to survey data, administrative registers have often been used throughout history, and in recent decades they have played a key role in the production of official statistics [53, 63]. However, the frontier of data integration and inference relates to three relatively new data sources: volunteer web surveys, big or digital trace data, and mobile data collection [29].

Volunteer web surveys and opt-in panels were developed during the second half of the 1990s but gained popularity only 10 years later [7, 14], especially for market research and public opinion studies. Even though hundreds (or thousands) of questionnaires can be filled out online in a relatively short time, concerns remain about the generalizability of the results to the general population due to the self-selection of individuals [12]. As a result, several methodologies have been developed to address coverage and selectivity issues.

Big or digital trace data are defined as digital data generated by human interaction and systems (e.g., sensor data, social media, google trends, transactions, etc.). They are not generated for statistical purposes (also known as organic data, see Groves [43]), but they can allow for measuring new phenomena [83]. Since 2010, they have become increasingly popular in social science, mainly due to the diffusion of social media, which are particularly relevant to better understanding attitudes and behaviors [25, 46]. Also, statistical institutes are engaged in the production of experimental statistics based on big data [32]. There are, however, selectivity and measurement issues that cannot be ignored, as demonstrated by the Google Flu experiment, which initially appeared promising but then failed to predict outbreaks [55].

Finally, mobile data collection is directly linked to big data and developed in the last few years. Mobile surveys involve filling out surveys on, for example, tablets and smartphones, and collecting data using devices’ sensors (e.g., photos, geolocation sensors, accelerometer, etc.). A benefit of sensor data is that it potentially provides objective data free from errors commonly associated with self-reports [85]. However, participation is voluntary and individuals decide whether and which data to share [86].

Despite their differences, all three sources share the property of not being probabilistic. Nevertheless, given the variety of these data, the three statistical challenges (inference in presence of selection bias, measurement issues and quality aspects) described in Sect. 1 need to be addressed separately. Although the literature in this field is expanding rapidly, it is still limited. The following paragraphs present some of the studies addressing such issues.

Amaya et al. [2] and Sen et al. [82] explain how the Total Error Framework can be adapted to different big data sources. As for social media data, Salvatore et al. [75] present a quality framework for Twitter data, while Amaya et al. [1] address statistical issues related to Reddit data. An error framework for web-tracking data is presented by Bosch Jover and Revilla [49]. The opportunities and challenges associated with supplementing survey data with data from sensors and applications are discussed by Struminskaya et al. [85].

Issues in representation and measurement when augmenting surveys with auxiliary data are addressed by Stier et al. [83] and Braun and Kuljanin [19]. Einarsson et al. [38] and Baker et al. [7] also discuss measurement errors and mode effects in the context of online opt-in panels.

Despite the limited literature about data quality, error frameworks, and construct measurement, several studies focus on statistical inference in the presence of selection bias. Many traditional review articles have discussed the use of different inferential approaches to correct selection bias and integrate multi-source data. A comprehensive review of inference for non-probability samples has been published, for the first time, by Baker et al. [8]. In addition to reviewing the various non-probability sampling techniques, they also cover estimation and weight adjustment methods as well as considerations concerning the quality of the data.

Considering both missing-at-random (MAR) and missing-not-at-random (MNAR) selection mechanisms, Elliott and Valliant [40] describe three methods of estimation from non-probability samples: quasi-randomization, superpopulation modeling, and doubly robust estimation. The authors provide a discussion of the respective advantages and disadvantages. The effectiveness of such approaches is then examined through the use of a simulation study in Valliant [89].

Rao [70] and Beaumont and Rao [10] also review estimation methods, emphasizing data integration and demonstrating how big data can enhance small area estimation. Finally, Cornesse et al. [27] review the empirical evidence of using NPS for inference, suggesting under which conditions it is possible to obtain the highest accuracy. More recently, review studies focused on machine learning and bayesian methods for data integration [20, 58, 88].

The themes discussed above are expected to emerge from our analysis, as well as new insights regarding thematic evolution, potential applications and new research areas. The following section provides a detailed description of the research objectives.

## Research objectives and data

### Research objectives

In contrast to the previous studies, this article offers an alternative and original perspective and situates itself within the discipline of science mapping. We consider a larger number of publications and, using bibliometric and text mining techniques, we are able to map the literature, providing an updated *big picture* of the field in terms of the research community and topics development. A comprehensive longitudinal analysis is conducted to identify research patterns and trends.

In particular, this study addresses the following research objectives (RO):

*RO1.* To understand the annual growth of the scientific production.

*RO2.* To identify the most productive authors, the driving research groups, the leading outlets for publication, and in which topics authors are specialized (performance and social structure).

*RO3.* To explore the conceptual structure of the field.

*RO4.* To understand the evolution of the conceptual structure over the years (thematic evolution).

Based on the results of our analysis, we identify the research gaps and the emerging topics. Thus, the ultimate goal of the study is the following:

*RO5.* To outline and provide practitioners with a research agenda for future investigations.

### Data

Bibliographic information can be retrieved from various databases, including Scopus, Web of Science (WoS), and Google Scholar. We consider the Scopus database. Compared to WoS, it has a more comprehensive list of publications. Further, it provides search and API tools for extracting data, resulting in higher quality data than Google Scholar, which is the most extensive database. Also, Google Scholar does not allow to define as specific and advanced search queries compared to Scopus and WoS.

A two-step retrieval strategy is used. First, a search query is formulated in order to retrieve publications about methodologies for data integration and statistical analysis of non-probability samples. The resulting list of documents is manually inspected in order to remove out-of-scope publications and keep and only topic-relevant documents. We refer to them as *seed* publications. Secondly, the dataset is expanded by selecting both cited and citing documents. This selection strategy aims to maximize the topic relevance and time coverage. In this way, the selected dataset’s analysis should mirror the field’s development.

The search query is based on the presence of keywords in the title and abstract, plus restrictions on language and subject area. Only publications (journal articles, conference proceedings, books, etc.) written in English and in the Mathematical field are considered. Appendix A discusses the keywords used to extract the publications in greater detail. Such keywords are identified based on the conceptual background outlined in Sect. 2.

**Fig. 1:**
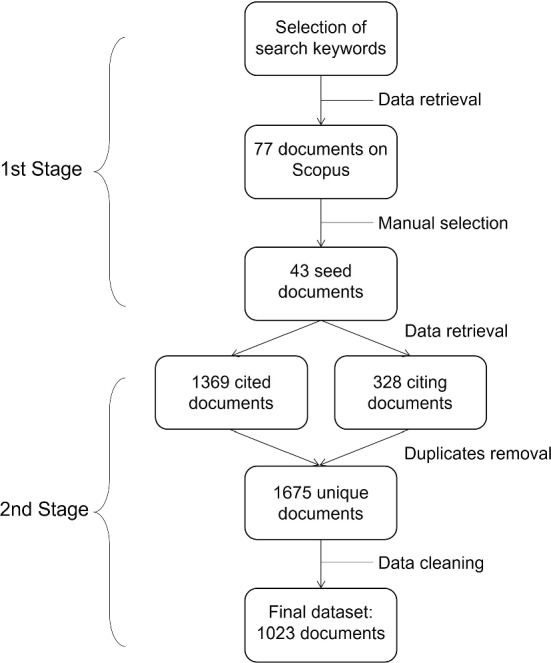
Data selection strategy

The number of papers extracted by the query is 77, out of which 43 are considered as *seed* publications. With the inclusion of cited and citing publications, the full dataset accounts for 1675 items. However, we restrict our analysis to documents for which the title, abstract, year, outlet and author’s identifiers are available. Thus, the final dataset contains 1023 publications. Figure 1 describes the data selection strategy and the cleaning progcess. Research papers are the prevalent document category (82%), followed by review papers (8%), books and book chapters (7%), and conference papers (3%).

In terms of authorship, 17% of documents are single-authored, 30% have two authors, 23% have three authors, and the remaining 30% have four or more authors. The publication years range from 1937 to the present.

## Methods

Bibliometric analysis entails analyzing scientific publications and their metadata using statistics and text mining. Using such methodologies allows for the assessment of citations, field growth, conceptual structure, leading authors, trends, and scientific communities [36]. Bibliometrics has proven to be a valuable tool for providing a comprehensive overview of journals [5, 37] or research fields [11, 31, 76].

A typical bibliometric study employs two main approaches. The first is performance analysis, which refers to the study of the authors’ and journals’ performance and co-citation analysis [62]. The second is science mapping, which aims to identify the domain’s structure in terms of topics and their evolution [17, 65]. In both cases, statistical methods are used, including text mining, clustering, and, most importantly, network analysis. For an introduction to bibliometric analysis and methodologies please refer to Noyons et al. [64] and Aria and Cuccurullo [4].

Specifically, we use network tools to investigate both the social and conceptual structure (RO2–3–4). In the former case, collaboration networks among authors and countries are provided [68]. In the latter, the co-words network is considered to identify clusters in topics and study their longitudinal evolution in the pre-defined subperiods [23]. Themes are identified, in each subperiod, using a community detection algorithm named *walktrap* on the co-occurrence matrix of terms [54]. Then, the results can be plotted using the thematic diagram [26]. It is a Cartesian plane where Callon’s centrality is on the *x*-axis, and Callon’s density is on the *y*-axis [22].

The Centrality measures the interaction between networks (topics). Thus, it indicates the relative importance of a topic within the collection of documents. The density measures the strength of internal links among the terms describing the topic. Essentially, it is a measure of the topic’s development. According to these definitions, each quadrant of the cartesian plane can be read as a different *theme typology*. In the upper-right quadrant, there are *motor-themes* which are both well developed and important in the field. On the upper-left side are the *niche-themes*, which are well developed but not strongly associated with other themes. *Emerging* or *disappearing themes* are in the lower-left quadrant (characterized by low centrality and low density). In the last quadrant, there are *transversal* and *basic themes*, which are well connected with most of the themes. In addition, a preliminary assessment of thematic evolution can be made by examining the word dynamics. It entails analyzing the popularity of terms (e.g., unigrams, bigrams etc.) in titles, abstracts or keywords list over the years.

Lastly, text mining tools are necessary to clean and prepare the data. It is especially important to clean abstracts since some of them include the journal’s name and copyright symbols or follow a specific format divided into subsections (e.g., Introduction: [...], Motivation: [...], Results: [...]). Such structures are eliminated together with stopwords. Words are also singularized. We mainly consider the document’s abstract for analyses, which provides a greater level of detail with respect to short titles. We also analyze keywords but only for preliminary analyses, which are only available for 783 documents.

To summarize the methods, Table 1 shows, for each of the research objectives, the methodology associated with it.

**Table 1:** Research objectives and relative methodology

RO#	Objective	Methods/approaches
RO1	Temporal evolution	- Time series plot
RO2	Performance and social structure	- Authors networks
		- Three-fields plot (Sankey diagram)
RO3	Conceptual structure (CS)	- Co-words network analysis
		(Abstract and Keywords)
RO4	Thematic evolution (CS)	- Word dynamics (Keywords)
		- Thematic evolution map
RO5	Research agenda	- Qualitative approach (global evaluation of research themes)

In order to perform the analysis, we use the “bibliometrix” R package [4]. It allows to perform bibliometric analysis directly in R or using the accompanying interactive Shiny app.

## Results and discussion

### RO1: field development

Even though the field of survey data integration and inference for non-probability samples is still relatively new, our data retrieval strategy allows us to go back in time, providing a general perspective on the evolution of that field. As a matter of fact, the first paper in the dataset was published in 1937, and it is about the Straw election polls [30].

Based on the 1023 documents published from 1937 to 2022, Fig. 2 shows the year-wise distribution for the full and selected (clean) datasets following the procedures described in Sect. 3.2. Although 643 publications are excluded due to the absence of relevant information (Authors, Title, Abstract, Source, and Year), the two curves exhibit similar characteristics.

Prior to the 1990s, the number of publications is constant and low. Following the discussion about the conceptual background of this study, we expect this period to be characterized by fundamental papers dealing with general statistical methodologies, nonresponse, and polls.

**Fig. 2:**
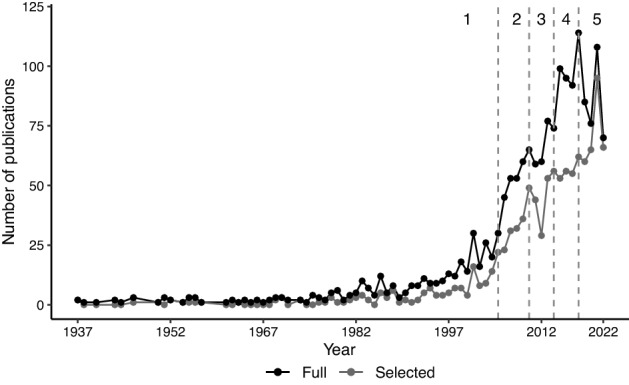
Year-wise distribution of publications in the full (black) and selected (grey) datasets. The five subperiods are indicated on top

Starting from the late 1990s, the number of publications increases, especially after 2005. Indeed, this is a very dynamic period characterized by the advent of big data and new data sources. We expect to have more insights through the thematic analysis. For this purpose, we consider five subperiods, which are shown in Fig. 2 (1937–2005; 2006–2010, 2011–2015; 2016–2019; 2020–2022). The first subperiod, 1937–2005, covers the early developments in the field. For a more in-depth understanding of recent developments and to capture the dynamicity of the research field, the following subperiods cover approximately 5 years each. These partitions should allow to identify trends in research with a good level of detail. Indeed, considering the analysis of the conceptual background, we expect each period to be characterized by the rise of novel data sources, new statistical challenges and methodological advances. The period 2006–2010 should be characterized by an expansion of the web as a tool for data collection and an increased use of administrative data, as outlined in Sect.  2. After 2010, we expect the rise of new (digital trace) data sources as well as discussions regarding opportunities and challenges associated with the use of such data. A specific subperiod is assigned to the 3 years of the coronavirus pandemic (2020–2022).

Table 2 shows the number of documents for each subperiod in the full and selected (clean) data sets. As a result of the temporal division, each subperiod also has a similar number of documents.

**Table 2:** Number of publications by subperiod in the full and selected datasets

Subperiod	No. of publications (selected)
1937–2005	366 (169)
2006–2010	276 (171)
2011–2015	369 (232)
2016–2019	386 (228)
2020–2022	254 (224)

Regarding *RO1*, it is evident that what was once a relatively young field has experienced rapid growth in recent years. Starting from 2010, the number of publications grew significantly. This growth can be explained and is aligned with the conceptual background (Sect. 2). From that year onward, web surveys became increasingly popular, and new data sources (e.g., big data and mobile data collection) became available.

### RO2: performance and social structure

**Fig. 3:**
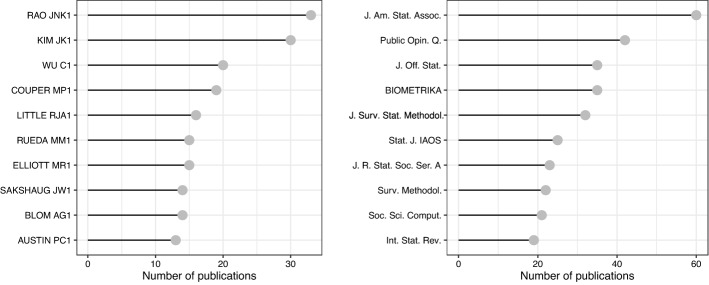
Top 10 authors and journals by number of publications

**Fig. 4:**
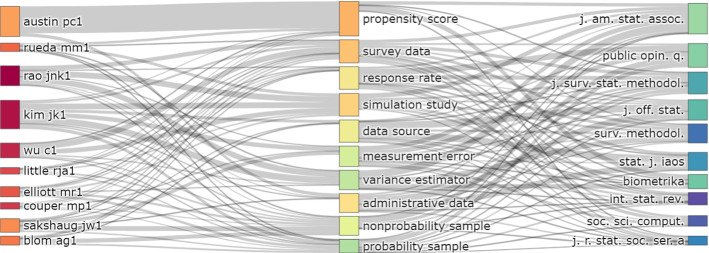
Three fields plot between authors, abstracts’ bigrams and publication outlet

To further characterize the scientific production, we consider authors, publications outlets and their link with main themes. Figure 3 shows the 10 most popular authors and publication outlets. It has been necessary to conduct a match between the names and identifiers of the authors in order to compensate for different formats and misspellings. Journals have been abbreviated according to the ISO-4 standard. Figure 4 links them with the 10 most popular bigrams in abstracts (i.e., two consecutive terms) by means of a Sankey diagram. It is a flow diagram and the width of the links corresponds to the flow rate. Authors are in the first column, bigrams in the second and publication outlets in the last one.

**Fig. 5:**
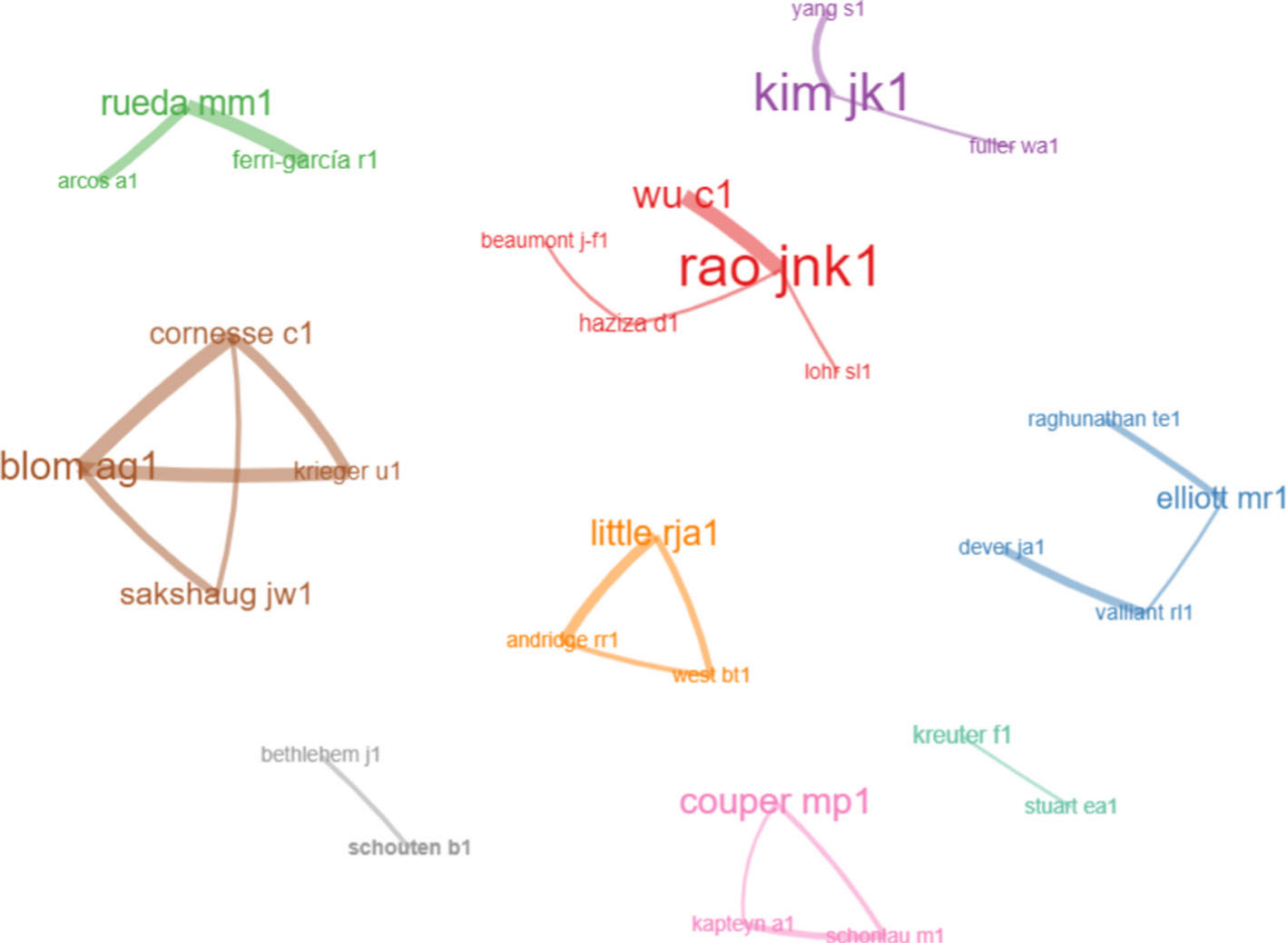
Author collaboration network

In terms of research groups, Fig. 5 shows the co-authorship network. In order to exclude one-off collaborations from the representation, the network analysis is based on the first 40 authors and restricted to those involved in at least two co-authored publications. Furthermore, the label size is proportional to the number of papers in the dataset, and the thickness of the edges, which indicate collaboration, is proportional to the number of co-authored papers. A total of nine driving research groups are identified. In order to gain a deeper understanding of the data, it is interesting to look at these three figures together.

Rao and Wu, the first and third top authors, are also part of the same cluster together with Haziza, Beaumont and Lohr. Their broad research topics mainly focus on survey weighting and the evaluation of inferential and data integration techniques using simulation studies. The second top author, Kim, collaborates with Yang and Fuller, considering a missing data perspective when analyzing NPS. Couper and his co-authors mainly address issues in web surveys and new data sources. The research group including Little, Andridge and West focuses primarily on selection bias and analytic inferences. Rueda and his co-authors focus on propensity score and calibration, while Elliot’s group focuses mainly on model-based approaches. The collaboration among Sakshaug, Blom, Cornesse, and Krieger focuses on studies examining measurement error, administrative data, and online panels. The network does not include Austin, which has mainly one-off collaborations with many authors and is involved with medical statistics. Finally, two additional small groups are identified. The first one includes Kreuter and Stuart, which consider the perspective of causal inference when addressing selectivity. The second one is made up of Bethlehem and Schouten, which focuses on nonresponse and selection bias.

In terms of the most popular publication outlets, the Journal of the American Statistical Association takes the lead. Based on Fig. 4, it is possible to identify bigrams (e.g., themes) that are distinctive to each journal, hence, identifying a polarity in themes discussed. For example, administrative data is primarily addressed by the Journal of Official Statistics and the Statistical Journal of the IAOS. Measurement error and response rates are specific to Public Opinion Quarterly and the Journal of Survey Statistics and Methodology. Studies about propensity scores or simulation studies are mainly published in the Journal of the American Statistical Association and Biometrika.

**Fig. 6:**
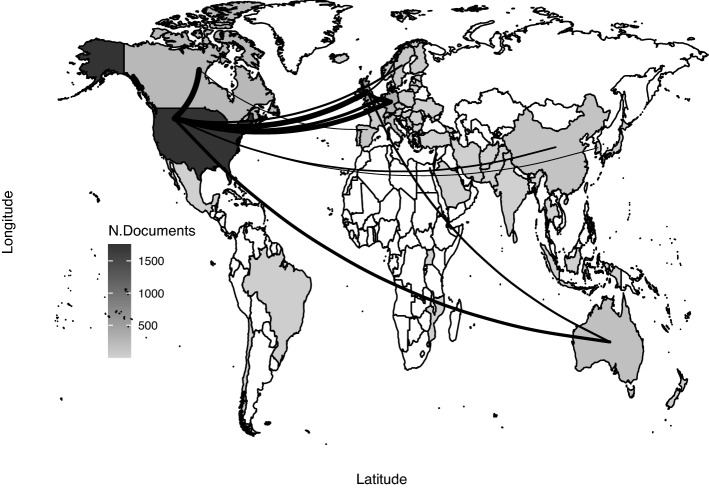
Country production by author affiliations and collaboration network (top 10)

In terms of country production and collaboration, it is possible to look at Fig. 6. The USA is the most productive country, followed by UK and Germany. The figure also shows the first ten collaboration edges, whose size is proportional to the number of co-authored documents. Major collaborations are evident between USA and other countries, primarily Canada, UK and Germany.

**Fig. 7:**
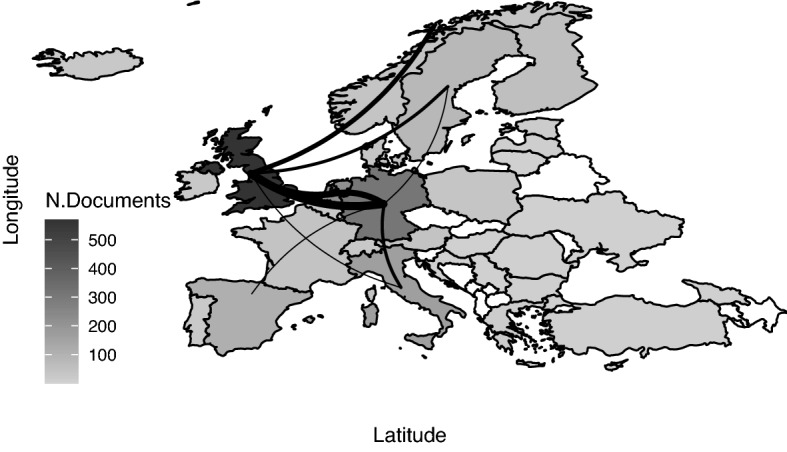
Country production by author affiliations and collaboration network (top 10 in Europe)

Figure 7 zooms in on European countries where the most productive and collaborative ones are UK, Germany, the Netherlands and Italy (with more than 150 publications each).

As for *RO2*, the analysis allowed us to determine which research groups are driving the research, which journals are the most influential, and how polarized the themes are within the field.

### RO3: conceptual structure

**Fig. 8:**
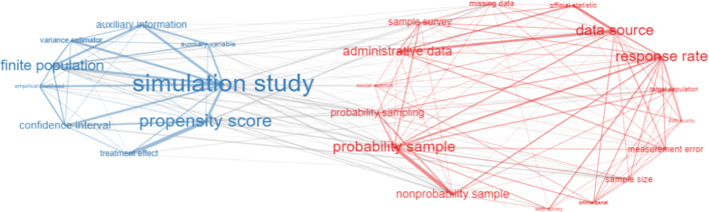
Abstracts’ bigrams co-occurrence network

**Fig. 9:**
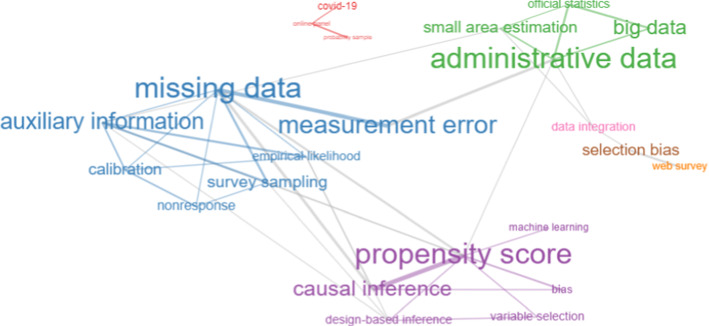
Keywords co-occurrence network

The conceptual structure of a field can be revealed through network analysis by mapping co-words. Indeed, each topic can be identified by a set of terms. Such terms are usually a set of keywords assigned by authors to their manuscripts or can be extracted from abstracts or titles. We consider bigrams extracted from abstracts which are more informative and descriptive than titles. Keywords are more distinctive of the document’s topic, while abstracts’ bigrams can help illustrate more details about studies. Therefore, we analyze both types of terms. The analysis of keywords is limited to 783 documents for which they are available. To have a static idea of the conceptual structure of the field, Figs. 8 and 9 show the co-occurrence network considering keywords and abstracts’ bigrams. The networks include the top 25 terms with at least two edges for both cases. Word clusters are characterized by different colors. The internal links between words within the same cluster have the same color. The gray color indicates external links between words that are assigned to different clusters but co-occur together in documents.

From the analysis of bigrams, it is possible to distinguish two main clusters. The first relates to different inferential methodologies (e.g., simulation study, propensity score, finite population, etc.). The second relates to substantive aspects such as the availability of new data sources which arose as a consequence of technological changes and their related issues (administrative data, PS and NPS, web survey/online panel, official statistics, measurement error, etc.). It is evident that there are many external (gray) links linking the two clusters, which indicates that they are highly interconnected. Keyword analysis also yields similar clusters related to methodological and practical aspects. In addition, there is also a society-related topic, the coronavirus pandemic. Indeed, online volunteer panels and social media-based surveys have been the subject of many social science studies concerning its impacts [78].

This analysis, even though static, provides a general idea of the main topics in the fields, addressing *RO3*. The next step is the study of the conceptual structure over time. It concerns the evolution of themes through six subperiods, as discussed in Sect. 5.1. We consider the same categorization as for the themes emerged in this static analysis (methodological, substantive and applied/society-related).

### RO4: thematic evolution

This section examines the conceptual structure of the field through thematic evolution analysis. Using this method, we can identify the topics and their evolution during the five time slices under consideration (1937–2005; 2006–2010; 2011–2015; 2016–2019; 2020–2022). Essentially, it involves representing the terms that appear together in a document as a term co-occurrence network and implementing a community detection algorithm (walktrap) in order to identify themes (see Sect. 4 for more details). In regard to terms, we consider abstracts’ bigrams that provide a good level of detail with respect to titles and keywords. In order to exclude infrequent bigrams, we restrict the analysis to those that appear in more than three documents, separately for each subperiod (which corresponds roughly to the 2% of documents). This is a common pre-processing step in text mining [34].

**Fig. 10:**
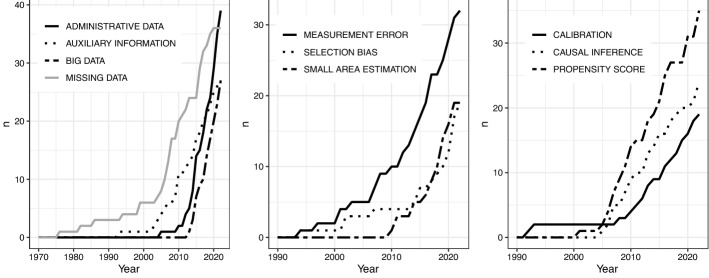
Top 10 keywords dynamics (cumulative frequency distribution)

However, before analyzing themes in greater detail, we focus our attention on keyword dynamics. Despite the fact that the analysis is limited to 783 documents, it does provide an overview of the most popular topics and their evolution over time. Figure 10 shows the cumulative frequency distribution for the top 10 keywords. Missing data is the first term appearing in the late 1970s. Indeed, the analysis of NPS can be approached as a missing data problem and the use of this keyword grew significantly from 2005 onward. Since the late 1990s, auxiliary information has been of interest to researchers. As an auxiliary data source to traditional surveys, administrative data (2005) and big data (2013) have emerged in recent years. On the other hand, classical statistical error issues (measurement error and selection bias) became more important and central in the methodological literature starting from 2010. Methods for data integration and inference using non-probability samples emerged as well, such as small area estimation, calibration, and propensity score (originally developed for causal inference). This dynamic is coherent with the conceptual background discussed in Sect. 2.

**Fig. 11:**
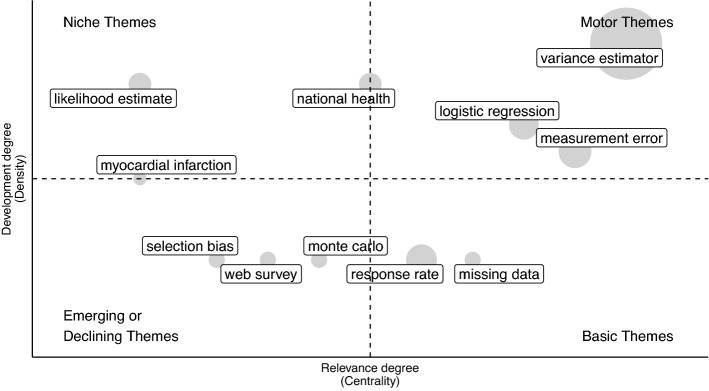
Thematic map 1937–2005

**Fig. 12:**
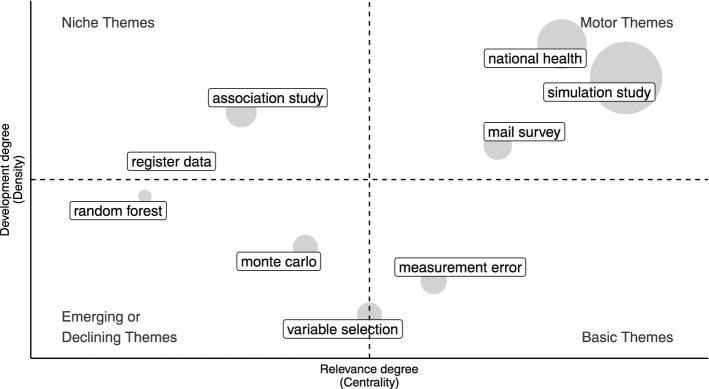
Thematic map 2006–2010

**Fig. 13:**
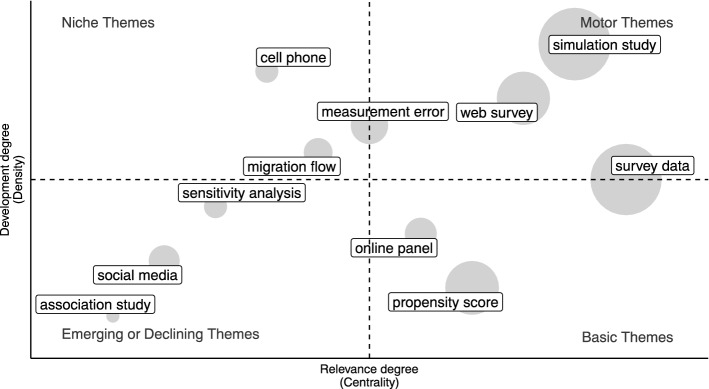
Thematic map 2011-2015

**Fig. 14:**
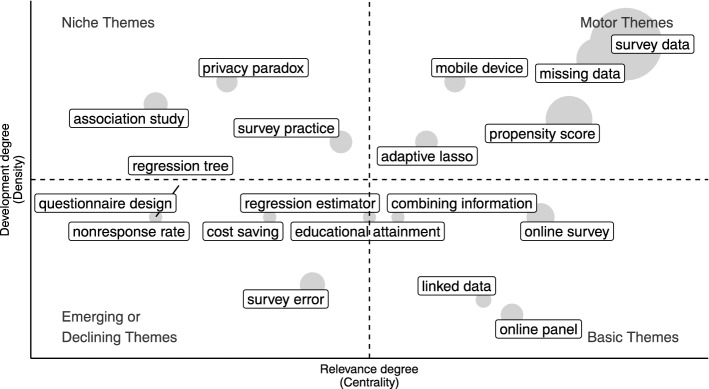
Thematic map 2016–2019

**Fig. 15:**
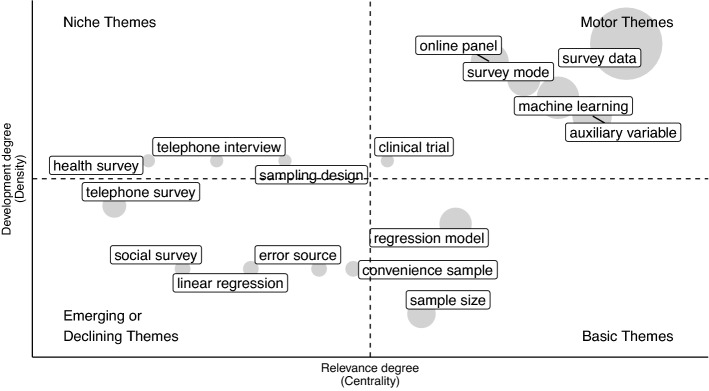
Thematic map 2020–2022

In order to gain further insight into themes in each subperiod, thematic maps can be constructed. The themes are sized in proportion to their importance in the collection of documents, and the most frequently occurring bigram is reported for each cluster (Figs. 11, 12, 13, 14, 15). When interpreting a cluster, we examine the documents most associated with it, along with other bigrams.

In this part, we adopt the same theme categorization as in the static conceptual structure analysis. Themes are classified in three categories of topics. The first one relates to methodological topics regarding inferential and data integration techniques (e.g. propensity score, variance estimation, regression analysis, etc.). The second one is about substantive topics that emerged as a consequence of technological innovation (e.g. register data, administrative data, online panel, social media, privacy paradox, linked data, etc.). The last class pertains to topics that reflect the research directions relevant to society and for which NPS data can be used (coronavirus pandemic, health care, educational attainment, etc.). The following subsections provide a detailed examination, organized according to the above-mentioned categories of topics, of each time slice. Detailed comments are provided only for the largest and most relevant clusters.

#### The first developments: 1937–2005

Prior to 2005 (Fig. 11), it is possible to identify the *methodological theory* (e.g. variance estimation, measurement error, missing data, likelihood estimate) which is at the core of new inferential and data integration techniques. Measurement error and variance estimation are motor themes, which means they are highly interconnected to other topics, as well as highly developed within the field.

Among *substantive topics* web surveys and selection bias emerges. The declining response rate is a basic theme, which means that it is generally studied in conjunction with other themes. For example, looking at associated documents, the relationship between selection bias, drop-out, and the response rate emerges, especially in relation to web surveys [16, 77, 80].

As part of *applied and society-related themes*, national health surveys and health registers are used to address migration and medical studies (myocardial infarction) [6, 81].

#### Administrative data and web surveys: 2006–2010

In the second period, the biggest cluster is about *methods studies*, for which the most frequent bigram is simulation analysis (Fig. 12).

Looking at other bigrams and associated documents to that cluster, there are studies about propensity score models to address selection bias, variance estimation, sampling, and response rates. In the majority of these studies, such issues are addressed in relation to web surveys. For example, Bethlehem [12] discusses self-selection and undercoverage in web surveys, and Schonlau et al. [79] and Lee and Valliant [56] address selection bias using the propensity score technique. Also, the statistical aspects of using administrative data in official statistics are discussed [90]. Measurement error, which was a motor theme in the previous time slice, becomes less developed in the literature and moves to the category of basic themes.

As *substantive themes*, we find again mail surveys which is now a motor theme, indicating that it is well developed and strongly interconnected with other topics. This is also evident from the analysis of methods themes. Additionally, such studies also compare incentive effects between face-to-face and web surveys [73].

Register data is an emerging topic that is connected to both methods and *applied* studies. For example, register data are used in the field of agriculture [24], demographic [3] and health-related statistical studies [69]. A niche theme related to applied topics is genome-wide association studies.

#### New (big) data sources: 2011–2015

In line with the conceptual background, after 2010, web surveys and online panels became viable alternatives/supplements to traditional surveys, and new big data sources emerged (Fig. 13).

Indeed, as *substantive topics*, social media is an emerging theme, especially with reference to the analysis of Twitter data, while online panels and web surveys are basic and motor themes, respectively. In particular, the literature addresses the mode effect when considering mixed-mode surveys [45] or when comparing probability and non-probability (online) surveys [41]. A connected theme is the cluster of “survey data” which contains bigrams related to new data sources, administrative data, official statistics, survey mode, and data quality. Indeed, the opportunity and the challenges of using big data in survey research and official statistics are discussed in many studies with particular reference to the quality of the data (see for example, Struijs et al. [84], Tam and Clarke [87], Kitchin [52]).

From a *methodological* point of view, the cluster related to simulation studies and methodologies for statistical inference is always a motor theme. The propensity score separates from this cluster and becomes a basic theme. In parallel, high dimensional propensity score methods emerge and applications are evaluated through sensitivity analysis [71]. The measurement error topic moves toward the direction of niche themes.

The main *applied topics* relate to genome-wide association studies (declining theme) and migration flows (niche theme). Besides these topics, also social media data are used to investigate various aspects, such as smoking behavior [61] and communication about palliative medicine and physical activity [66, 92].

#### Mobile devices, data integration and the privacy paradox: 2016–2019

The fourth period is very dynamic in terms of themes (Fig. 14). As for the *methodological literature*, we can still see the presence of propensity score and missing data, plus new clusters about regression estimator (model and design based inference), machine learning methods (regression tree), adaptive lasso, nonresponse rate and survey error.

The clusters of simulation studies, measurement error, and other methodologies merge with the cluster of survey data (which included administrative data, new data sources and official statistics). This new cluster reflects the temporal dynamics of topics. Although these *methods and substantive themes* have taken different paths in the past (emerging, niche, or basic themes), they are now very well integrated within each other and well developed in the literature. As a result, a mixed cluster is formed.

Within the *substantive themes*, online survey and panel take the position of basic themes, while mobile device and technology is one of the leading topics in the research (motor theme). Some studies discuss the opportunity of administering a questionnaire on smartphones or other mobile devices, and the differences in measurement and response rate between devices/modes [39, 59, 72]. An important related concept is the willingness of respondents to use mobile apps for surveys and sharing data [47, 50, 91]. As we move into the digital age, privacy concerns related to the donation of personal data are becoming more relevant. It is still a niche theme, and few authors discuss the privacy paradox, which refers to the discrepancy between what respondents claim and their actual behavior with regard to online behavior and personal data protection [9].

From a data integration perspective, the “combining information” cluster is a basic theme [51, 67]. Similarly, also the topic of linked data is a basic theme. The purpose of the technique is to combine information from different sources in order to develop a new, richer dataset [33]. In the literature, the cost-saving argument emerges as a rationale for integrating survey data and using new data sources. In fact, the objective of many studies is to develop methodologies that allow for inferences to be drawn, potentially resulting in cost savings [74].

The genome-wide association studies are still being studied as a part of *application themes*, but they have become a niche topic over the years. Due to the wide range of topics, no other specific application clusters emerge from this analysis.

#### Recent developments and the coronavirus pandemic: 2020–2022

In the last 3 years, the coronavirus pandemic has shaped the research, not only in terms of *applied research* (health and socio-economic impacts of the pandemic), but also in terms of data collection (*methods and substantive topics*).

Indeed, researchers were forced to change the method of collecting data from face-to-face surveys to either online data collection or telephone surveys (Fig. 15). An example from the “online panel” cluster is the transition from the German Internet Panel to the Mannheim Corona study. The objective was to adapt the infrastructure to collect daily data in order to provide practitioners with updated information to study the socio-economic effects of the pandemic [15, 28]. In this context, social media might also be relevant for administering surveys [18, 57]. The “coronavirus pandemic” is part of the survey data cluster, which is a motor theme. Similarly, also machine learning is a motor theme, which means that both topics are well developed and highly interconnected with other themes.

Considering the current scenario, in which several data sources are available and methodologies are being developed to address inferential aspects, the theme of error sources emerges [35].

The coronavirus pandemic made it clear the role of technology in survey research and the need to develop inferential frameworks and data integration techniques in order to make use of auxiliary data (digital trace, web surveys, passive data collection, and administrative data). It implies the study of different aspects, including measurement error, selection bias, different error sources, and new sampling strategies.

In order to gain a better understanding of how themes have evolved over time, addressing *RO4*, the thematic evolution analysis was performed taking into account the three categories of topics identified in the static conceptual structure. With respect to substantive and methodological research, a cyclical pattern has emerged. Many of the themes shifted between the four dimensions considered (emerging, niche, motor, and basic). It is important to note that substantive and methodological themes are also closely interconnected. As soon as a new data source is discovered and new opportunities are investigated, new methods are developed to address inferential aspects.

In terms of applied research, the themes revealed by our analysis are mainly related to health and medical studies. One possible reason is that large amounts of health registers and claims data are readily available, making methodological studies through simulation analysis easier. Besides educational attainment and migration flow studies, other massive socioeconomic topics do not emerge. It may also be due to the wide variety of aspects that do not constitute a singular topic. As a matter of fact, when reviewing documents, we find applications related to agriculture, demographics, psychology, and social statistics.

## Concluding remarks

### Main findings

A deep transformation is occurring in survey research with regard to the use and integration of new data sources for inference. The literature has been reviewed in many papers in light of methodological advancements, but a comprehensive study about the evolution of the field is lacking. In order to address this gap, we map the literature by providing a link between methodological, substantive, and applied themes. We employ an original approach that combines tools for bibliometric analysis and text mining in order to achieve this goal. In contrast to previous literature reviews, this study analyzes a greater number of papers in order to gain a deeper understanding of how research has evolved in response to changes in data sources and technology diffusion. This is crucial for identifying emerging trends for future research.

In particular, this paper provides an original contribution to the literature in two ways. Firstly, it characterizes the field of inference for NPS and survey data integration in terms of bibliometric performance and social structure (*RO1–3*). The leading research groups and the most productive authors are identified. Several collaborations between countries have emerged, primarily between the United States and Germany, and with reference to European countries, between United kingdom and Germany. There is also evidence of a polarity in the topics covered by journals.

Secondly, our study outlines the evolution of the field in terms of conceptual structure (*RO4*). The results of this analysis indicate that advances in survey research and technology are closely related topics. As a matter of fact, technology is both a tool and a driver of innovation. In our digital era, the research is becoming increasingly data-driven, so the need for a methodologically sound framework for inference is crucial. There is evidence of a cyclical pattern in the topic evolution across the four dimensions (emerging/declining, niche, motor, and basic) and in terms of topic typology. Indeed, new methodological aspects are investigated as soon as a new data source becomes available.

There are, however, a few points that should be discussed and clarified. Firstly, in our study only one source (Scopus) is considered. Although it is one of the largest bibliographic databases and provides high quality data, some results may be missing. The issue is, however, not of great concern. Indeed, in the scientometric literature, different sources have been compared and there is evidence of a high level of overlap between them [42, 44]. Secondly, the formulation of the query may affect the results (selectivity). To understand the extent of this issue, we performed a sensitivity analysis using different keywords and identified the query described in Appendix A. Thirdly, we do not consider publications that lack adequate information, as described in Sect. 3. As a result, there are fewer documents in the final collection. While we are aware of the points outlined above, we believe that they are not significant concerns. We believe that the study is valuable in explaining the main themes and their evolution. Indeed, our bibliometric analysis is consistent with the conceptual background described in Sect. 2. The results are coherent and allow a better understanding of the social and conceptual structure of the field.

As a conclusion to this paper, we address the last objective of the research. Thus, we identify gaps in the literature based on our analyses and we outline a research agenda for future investigations (*RO5*).

### A research agenda for future investigations

The thematic analysis of the field of survey data integration and inference for non-probability samples reveals that it has undergone significant changes in response to the rise of new data sources and the challenges they present. In general, we observe a shift from the early period of research, when most focus was placed on aspects related to traditional (interview-based) probability sample surveys, to new areas of research. This shift has been accelerated by the pandemic which has emphasized the need to innovate in survey research, making use of different survey modes, new data sources, and of non-traditional methods in survey methodology, like machine learning.

The transition from traditional interview surveys to telephone and web surveys is a long-standing trend in the field. Through the thematic analysis, we have observed an evolution in online surveys, starting with web and mail surveys and progressing to online (opt-in) panels and web surveys administered on mobile devices (e.g. smartphones, tablets). This transition has led to new considerations for questionnaire design, and further research is needed to understand how to optimally design and integrate surveys that are administered using different modes and devices.

The pandemic has also increased the need for timely statistics for real-time monitoring and understanding emerging social aspects. This has led to a greater use of volunteer-web surveys and alternative data sources, such as social media, which in turn has brought increased attention to inferential and data quality aspects. An emerging topic that requires further investigation is the classification of error sources in novel data sources. As data integration advances, it is also necessary to develop quality frameworks for evaluating combined products, and to understand how errors arise, accumulate, and interact throughout the entire process of inference and data integration.

With the use of digital trace data as an alternative or supplement to surveys, new privacy concerns have been raised. The ability to easily collect this data online or through donations from individuals has raised questions about the treatment of personal information and individuals’ willingness to share it. Similar to consent in surveys, individuals’ willingness to share their digital data (passive data collection) should be further investigated. The analysis of the literature reveals a contradiction between privacy concerns and actual online behavior (*privacy paradox*), which needs to be clarified.

Volunteer web-surveys and digital trace data share the same non-probabilistic nature. Thus, from a methodological perspective, the study of selectivity and the variables associated with it (selection or auxiliary variables) has been highlighted in the literature in recent years (Fig. 15). An open problem relates to the scenario where the selection mechanism is “missing not at random” (i.e., participation directly depends on the outcome variable of interest), which requires further research.

So far, statistical frameworks have primarily focused on the estimation of finite populations quantities. However, even analytic estimates (such as regression and correlation coefficients) are susceptible to selection bias. This direction has been rarely explored in the literature, and further developments are needed. As non-traditional methods in survey search, machine learning, in particular, is a topic that has gained significant attention in recent years (2016–2022), especially during the pandemic. It encompasses not only to the analysis of unstructured data, but also to the application of such algorithms to address classic survey methodology issues, including survey weighting, data integration and variable selection.

On the basis of our analysis, non-probabilistic data sources should not be viewed as substitutes for probability sample surveys, but rather as supplements to them. PS surveys are still the gold standard in research, and new technologies and data can help to address some practical issues (for example, nonresponse) and augment the information to gain a better understanding of the phenomena. This is coherent with other literature review studies [21, 27]. From our analysis, it appears clear that research in this field is moving towards the use of new data sources and survey modes. One key driver of this trend is cost savings (Fig. 14). Traditional PS surveys are facing challenges due to rising non-response rates and costs, making non-probability data a more cost-effective alternative. However, it is important to note that new inferential and data quality considerations must be taken into account when using non-probability data.

In conclusion, addressing the challenges and opportunities presented by non-probability data requires not only the development of methodological approaches, but also qualitative evaluations. For that reason, the collaboration between researchers from different research areas will be a key aspect for the development of the field.

## Appendix

### Search query

The search query has been selected after a sensitivity analysis considering different keywords. The objective is to select methodological papers about inferential-related topics and data integration with non-probability samples. The symbol “*” has the role of wildcards. For example “sampl*” returns both sample/samples and sampling. Plurals are considered internally by the search function. For more information about formulating search queries in Scopus, please refer to the Scopus Search Guide.^1^ The search query is made by four elements linked with the AND operator:

1. **TITLE**: “data integration” OR inference OR estimat* OR integrat* OR combin* OR compar* OR “selection bias” OR “self selection” OR selectivity OR representativ* OR “non probabili* sampl*” OR “nonprobabili* sampl*” OR “nonprobabili* survey*” OR “non probabili* survey*” OR “online panel*” OR “volunteer web survey*” OR “volunteer online survey*” OR “volunteer data” OR “nonprobabili* data” OR “non probabili* data” OR “smartphone survey*” OR “digital trace data” OR “administrative data” OR “mobile data” OR “self administ*”
2. **ABSTRACT**: (“data integration” OR inference OR integrat* OR combin* OR “selection bias” OR “self selection” OR selectivity ) AND (“non probabili* sampl*” OR “nonprobabili* sampl*” OR “nonprobabili* survey*” OR “non probabili* survey*” OR “online panel*” OR “volunteer web survey” OR “volunteer online survey” OR “volunteer data” OR “nonprobabili* data” OR “non probabili* data” OR “smartphone survey*” OR “digital trace data” OR “administrative data” OR “self administ*”)
3. **SUBJECT**: “MATH”
4. **LANGUAGE**: “English”
